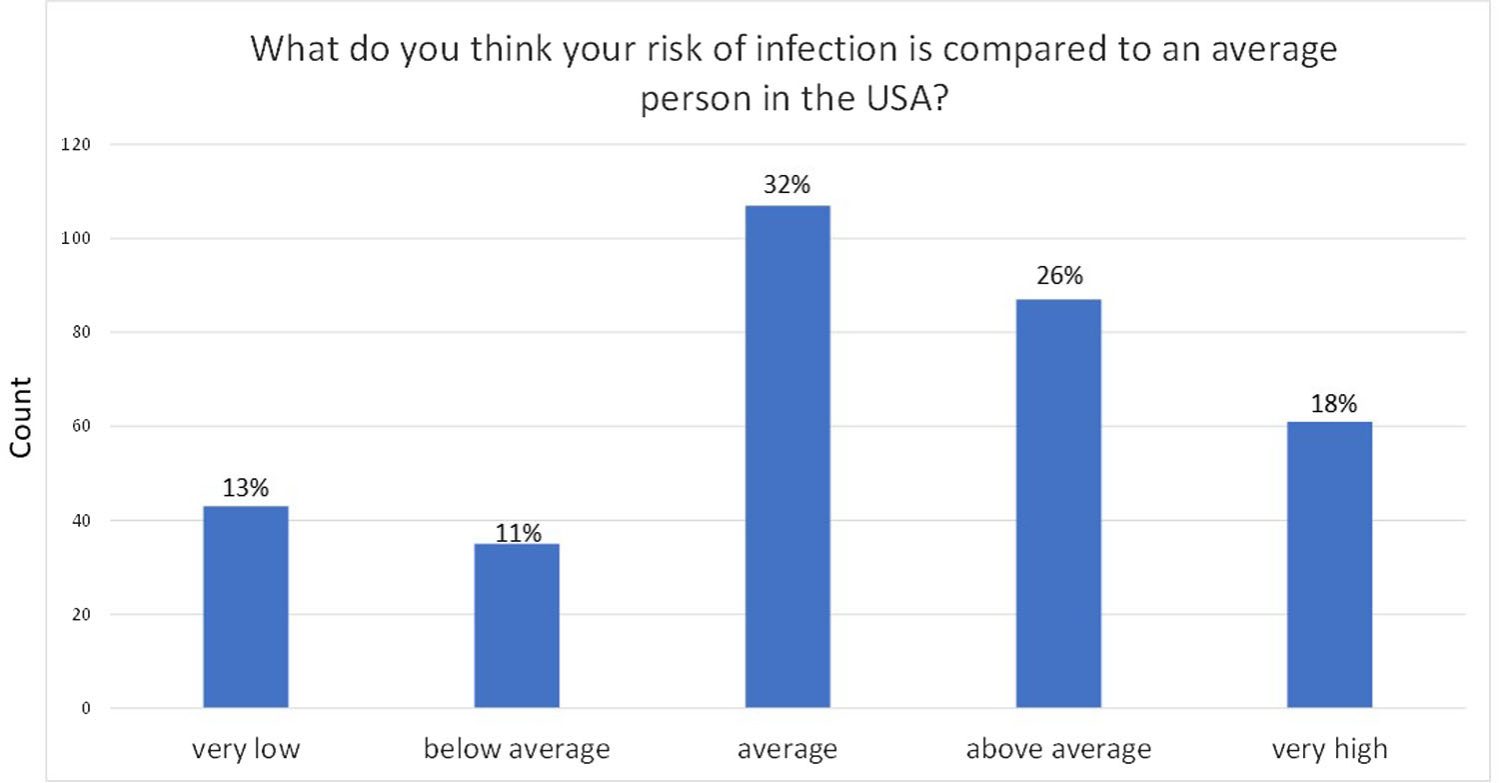# Survey of Hemodialysis Patients’ Knowledge of Their Infection Risk and Acceptability of a Nasal Decolonization Intervention

**DOI:** 10.1017/ash.2024.203

**Published:** 2024-09-16

**Authors:** Fiona Armstrong-Pavlik, AM Racila, Melissa Ward, Pam Tolomeo, Joseph Kellogg, Brenna Lindsey, Loreen Herwaldt, Rajeshwari Nair, Jesse Jacob, Anitha Vijayan, David Pegues, Jason Cobb, Mony Fraer, Susan Bleasdale, Kimberly Dukes, Stacey Hockett Sherlock, Marin Schweizer

**Affiliations:** University of Iowa Carver College of Medicine; University of Pennsylvania Perelman School of Medicine; Emory University School of Medicine; University of Illinois at Chicago; The University of Iowa; Emory University; Intermountain Kidney Services; Hospital of the University of Pennsylvania; Dept of Gen Int Med, Carver College of Medicine, University of Iowa; University of Wisconsin-Madison

## Abstract

**Background:** Patients undergoing hemodialysis are at high risk for healthcare-associated infections; they are at 100 times the risk of Staphylococcus aureus bloodstream infections (BSI) compared with U.S. adults not on hemodialysis. Prior studies found that nasal decolonization with mupirocin prevented S. aureus BSI among hemodialysis patients. We implemented a nasal decolonization intervention in which patients self-administered povidone-iodine (PVI) at each dialysis session. We aimed to assess: 1) hemodialysis patients’ knowledge of their infection risk and their willingness to take an active role in infection prevention; 2) the acceptability of the PVI nasal decolonization intervention. **Methods:** We performed a stepped wedge cluster randomized trial at 16 outpatient hemodialysis centers. Patients were surveyed: before starting PVI, 1 month after their center started using PVI, and ~6 months after starting PVI. We used a chi-square test to compare results. **Results:** 469 patients completed at least 1 survey: 400 pre-intervention, 237 at 1 month and 201 at 6 months. Overall, 56% of patients thought that their risk of infection was average or below average compared with an average person in the U.S. (Figure). Over 98% agreed with the statement “One of the most important things I can do for my health is to take an active role in my health care." In the pre-intervention survey, 73% were willing to do “a lot of effort” to prevent an infection. This proportion was similar (73%) in the 2nd survey, but decreased to 63% in the final survey (p < 0 .01). Among 106 patients who reported starting PVI, 85% reported that PVI felt neutral or pleasant, 9.4% reported a side effect, and 79% reported using it during the past 3 dialysis sessions. Among 102 patients who reported using PVI at 6 months, 87% said it felt neutral/pleasant, 3.9% reported a side effect and 75% reported using it during the past 3 dialysis sessions. Side effects included nasal dripping, congestion or burning/stinging, unpleasant smell, headache, yellow tears, and minor nose bleeding. **Conclusions:** Hemodialysis patients are not aware of their high risk of infection. Although many were willing to expend a lot of effort to prevent an infection, this willingness decreased during an infection prevention intervention. There were few PVI side effects and most patients stated that PVI felt neutral/pleasant, yet many patients chose to not use PVI. Future research should aim to improve patient education on their risk of infection and assess barriers to adherence with infection prevention interventions.

**Disclosure:** Marin Schweizer: Speaker- 3M; Contracted research-3M; Anitha Vijayan: Honoraria - Quanta, Baxter, Fresenius Consulting, Astute, NxStage

**Figure:**